# [2-(3,4-Dimethyl­benzo­yl)phen­yl](4-meth­oxy­phen­yl)methanone

**DOI:** 10.1107/S1600536812047654

**Published:** 2012-12-05

**Authors:** G. Jagadeesan, K. Sethusankar, R. Sivasakthikumaran, Arasambattu K. Mohanakrishnan

**Affiliations:** a Department of Physics, Meenakshi College of Engineering, West K.K. Nagar, Chennai 600 078, India; b Department of Physics, RKM Vivekananda College (Autonomous), Chennai 600 004, India; c Department of Organic Chemistry, University of Madras, Maraimalai Campus, Chennai 600 025, India

## Abstract

The title mol­ecule, C_23_H_20_O_3_, is disordered with a 180° rotation about an axis normal to the length of the mol­ecule, with the major and minor components in a 0.545 (5):0.455 (5) ratio. In the major component, the central benzene ring forms dihedral angles of 72.34 (3) and 69.46 (3)° with the dimethyl-substituted and meth­oxy-substituted benzene rings, respectively. Moreover, the central benzene ring forms dihedral angles of 50.86 (5) and 58.43 (4)° with the mean planes of the ketone groups. In the minor component, the corresponding dihedral angles between the benzene rings are 71.36 (4) and 67.94 (4)° and the dihedral angles between the benzene ring and the ketone groups are 56.44 (9) and 55.51 (8)°. In the crystal, C—H⋯O inter­actions generate a *C*(9) chain along the *a-*axis direction.

## Related literature

For the uses and biological importance of diketones, see: Sugawara *et al.* (2001 ▶). For the synthesis of heterocyclic compounds, see: Hirsch & Bailey (1978 ▶). For a related structure, see: Jagadeesan *et al.* (2011 ▶).
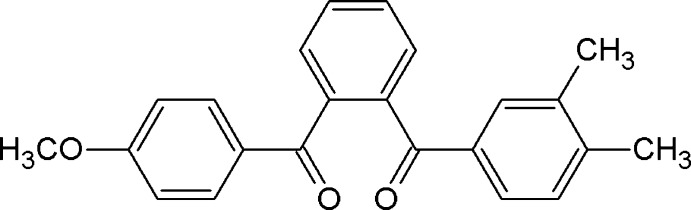


## Experimental

### 

#### Crystal data

C_23_H_20_O_3_
*M* *_r_* = 344.39
Monoclinic, 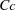

*a* = 21.983 (2) Å
*b* = 7.8173 (6) Å
*c* = 11.7489 (10) Å
β = 116.158 (2)°
*V* = 1812.2 (3) Å^3^
*Z* = 4
Mo *K*α radiation
μ = 0.08 mm^−1^
*T* = 296 K
0.35 × 0.30 × 0.25 mm

#### Data collection

Bruker Kappa APEXII CCD diffractometer
Absorption correction: multi-scan (*SADABS*; Bruker, 2008 ▶) *T*_min_ = 0.972, *T*_max_ = 0.980
9619 measured reflections
4759 independent reflections
2728 reflections with *I* > 2σ(*I*)
*R*_int_ = 0.021

#### Refinement

*R*[*F* ^2^ > 2σ(*F* ^2^)] = 0.051
*wR*(*F* ^2^) = 0.173
*S* = 1.02
4759 reflections
388 parameters
353 restraints
H-atom parameters constrained
Δρ_max_ = 0.25 e Å^−3^
Δρ_min_ = −0.16 e Å^−3^

### 

Data collection: *APEX2* (Bruker, 2008 ▶); cell refinement: *SAINT* (Bruker, 2008 ▶); data reduction: *SAINT*; program(s) used to solve structure: *SHELXS97* (Sheldrick, 2008 ▶); program(s) used to refine structure: *SHELXL97* (Sheldrick, 2008 ▶); molecular graphics: *ORTEP-3* (Farrugia, 2012 ▶); software used to prepare material for publication: *SHELXL97* and *PLATON* (Spek, 2009 ▶).

## Supplementary Material

Crystal structure: contains datablock(s) global, I. DOI: 10.1107/S1600536812047654/pv2606sup1.cif

Structure factors: contains datablock(s) I. DOI: 10.1107/S1600536812047654/pv2606Isup2.hkl

Supplementary material file. DOI: 10.1107/S1600536812047654/pv2606Isup3.cml

Additional supplementary materials:  crystallographic information; 3D view; checkCIF report

## Figures and Tables

**Table 1:** Hydrogen-bond geometry (Å, °)

*D*—H⋯*A*	*D*—H	H⋯*A*	*D*⋯*A*	*D*—H⋯*A*
C23—H23*B*⋯O2^i^	0.96	2.32	3.23 (3)	159

Symmetry code: (i) 
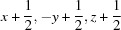
.

**Fig. 1.**
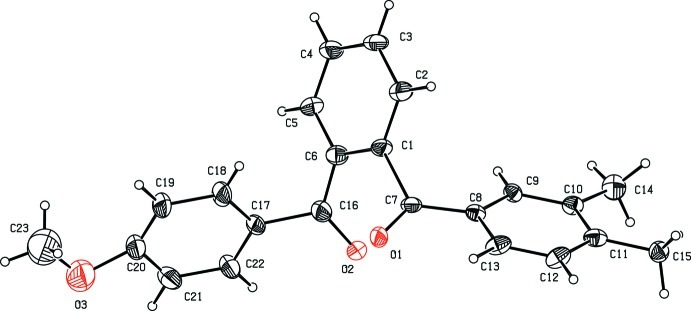
The molecular structure of the title compound (major component) with the atomic numbering scheme and displacement ellipsoids drawn at 30% probability level.

**Fig. 2.**
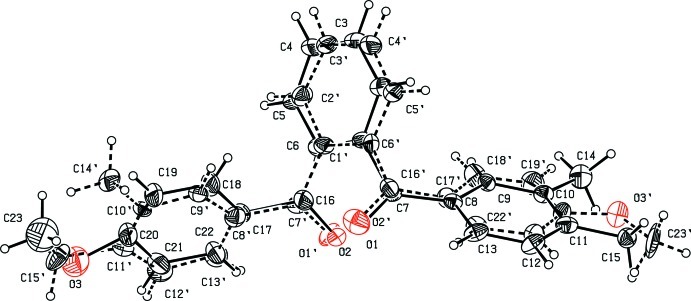
The molecular structure of the title compound with the atomic numbering scheme and displacement ellipsoids at the 30% probability level. The unprimed atoms represent the major component where as the minor component is represented by primed ones.

**Fig. 3.**
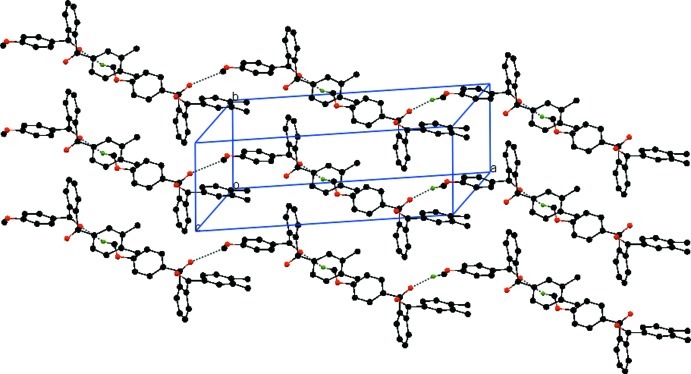
The crystal packing of the title compound viewed down c axis, dashed lines indicates C–H···O interactions. H–atoms not involved in hydrogen bonds have been excluded for clarity.

## supplementary crystallographic information

### Comment

The cyclic ketones play a significant role in increasing the red blood cells. They are also useful as hematopoietic agents in medicine, in particular, in the treatment of cancer, chemotherapy, radiotherapy and drug therapy (Sugawara *et al.*, 2001). They are also important synthetic intermediates and starting materials in the synthesis of heterocyclic compounds (Hirsch & Bailey, 1978).

The molecular structure of the title compound is shown in Fig. 1. The molecule is disordered with 180° rotation about an axis normal to the length of the molecule. The unprimed atoms represent the major component where as the minor component is represented by primed ones. The site occupancy factors of the major and minor components refined to 0.545 (5) and 0.455 (5) values, respectively. The disorder prevents a discussion of accurate molecular geometry, though values are comparable to those reported in a closely related methanone derivative (Jagadeesan *et al.*, 2011). The two overlapping molecules are shown in Fig. 2. There is a small separation between the coordinates of each pair of atoms. However, the atomic positions of all atoms in the two molecules are well resolved.

In the major component, the central benzene ring (C1–C6) forms dihedral angles of 72.34 (3) and 69.46 (3)° with the dimethyl substituted benzene ring (C8–C13) and methoxy substituted benzene ring (C17–22), respectively. Moreover, the benzene ring (C1–C6) forms dihedral angles of 50.86 (5) and 58.43 (4)° with the mean planes of the ketone groups (C1/C7/C8/O1) and (C6/C16/C17/O2), respectively. In the minor component, the central benzene ring (C1'–C6') forms dihedral angles of 71.36 (4) and 67.94 (4)° with the dimethyl substituted benzene ring (C8'–C13') and methoxy substituted benzene ring (C17'–C22'), respectively. Furthermore, the benzene ring (C1'–C6') forms dihedral angles of 56.44 (9) and 55.51 (8)° with the mean planes of the ketone groups (C1'/C7'/C8'/O1') and (C6'/C16'/C17'/O2'), respectively. The atoms C14 and O1 deviate significantly (-0.1305 (1) and -0.6096 (1) Å, respectively), from the mean plane of the benzene ring (C8–C13) and the atom O2 deviates by -0.6273 (2) Å from the mean plane of the methoxy substituted benzene ring (C17–22). On the other hand, the atom O1' deviates by -0.3678 (3) Å from the mean plane of the benzene ring (C8'–C13') and the atom O2' deviates by -0.3507 (2) Å from the mean plane of the methoxy substituted benzene ring (C17'–22').

The crystal packing is stabilized by C23–H23B···O2^i^ intermolecular interactions (Tab. 1) generating a *C*(9) chain along the *a* axis (^i^: *x* + 1/2, *y* - 1/2, *z* + 1/2). The packing diagram of the crystal structure is shown in (Fig. 3).

### Experimental

To a stirred suspension of [2-(3,4-dimethylbenzoyl)phenyl](4-methoxyphenyl) methanone (1 g, 3.22 mmol) in dry THF (20 ml), lead tetraaccetate (1.52 g, 3.42 mmol) was added and refluxed at 343 K for half an hour. The reaction mixture was then poured into water (200 ml) and extracted with ethyl acetate (2*x*20 ml), washed with brine solution and dried (Na_2_SO_4_). The removal of solvent *in vacuo* afforded crude product. The crude product upon crystallization from methanol furnished the tittle compound as a colorless solid.

### Refinement

The site occupancy factors of the major (unprimed atoms) and minor (primed atoms)components refined to 0.545 (5) and 0.455 (5) values, respectively. The approximate atomic positions of the disordered components were obtained from the difference electron density maps and the molecules were refined using suitable restraints. The benzene rings were refined as rigid hexagons with C–C distances 1.39 Å. The other bond-length of the major and the minor components were made similar using similarity restraints with s.u. of 0.01 Å. The atomic displacement parameters of each atom were made similar to the neighbouring atoms with suitable similarity restraints with s.u. of 0.01. Hydrogen atoms were placed in calculated positions with C–H = 0.93 and 0.96 Å for aryl and methyl type H-atoms and refined in the riding model with isotropic displacement parameters with *U*_iso_(H) = 1.5 *U*_eq_(methyl-C) or 1.2 *U*_eq_(aryl-C). Due to lack of sufficient anomalous dispersion effects in diffraction measurements on the crystal, an absolute structure was not determined; 2307 Friedel pairs were merged.

### Crystal data

**Table:**

C_23_H_20_O_3_	*F*(000) = 728
*M**_r_* = 344.39	*D*_x_ = 1.262 Mg m^−^^3^
Monoclinic, *C**c*	Mo *K*α radiation, λ = 0.71073 Å
Hall symbol: C -2yc	Cell parameters from 4759 reflections
*a* = 21.983 (2) Å	θ = 2.8–29.2°
*b* = 7.8173 (6) Å	µ = 0.08 mm^−^^1^
*c* = 11.7489 (10) Å	*T* = 296 K
β = 116.158 (2)°	Block, colourless
*V* = 1812.2 (3) Å^3^	0.35 × 0.30 × 0.25 mm
*Z* = 4	

### Data collection

**Table:**

Bruker Kappa APEXII CCD diffractometer	4759 independent reflections
Radiation source: fine-focus sealed tube	2728 reflections with *I* > 2σ(*I*)
Graphite monochromator	*R*_int_ = 0.021
ω scans	θ_max_ = 29.2°, θ_min_ = 2.8°
Absorption correction: multi-scan (*SADABS*; Bruker, 2008)	*h* = −30→30
*T*_min_ = 0.972, *T*_max_ = 0.980	*k* = −8→10
9619 measured reflections	*l* = −16→16

### Refinement

**Table:**

Refinement on *F*^2^	Primary atom site location: structure-invariant direct methods
Least-squares matrix: full	Secondary atom site location: difference Fourier map
*R*[*F*^2^ > 2σ(*F*^2^)] = 0.051	Hydrogen site location: inferred from neighbouring sites
*wR*(*F*^2^) = 0.173	H-atom parameters constrained
*S* = 1.02	*w* = 1/[σ^2^(*F*_o_^2^) + (0.073*P*)^2^ + 0.6313*P*] where *P* = (*F*_o_^2^ + 2*F*_c_^2^)/3
4759 reflections	(Δ/σ)_max_ < 0.001
388 parameters	Δρ_max_ = 0.25 e Å^−^^3^
353 restraints	Δρ_min_ = −0.16 e Å^−^^3^

### Special details

**Table:**

Geometry. All e.s.d.'s (except the e.s.d. in the dihedral angle between two l.s. planes) are estimated using the full covariance matrix. The cell e.s.d.'s are taken into account individually in the estimation of e.s.d.'s in distances, angles and torsion angles; correlations between e.s.d.'s in cell parameters are only used when they are defined by crystal symmetry. An approximate (isotropic) treatment of cell e.s.d.'s is used for estimating e.s.d.'s involving l.s. planes.
Refinement. Refinement of *F*^2^ against ALL reflections. The weighted *R*-factor *wR* and goodness of fit *S* are based on *F*^2^, conventional *R*-factors *R* are based on *F*, with *F* set to zero for negative *F*^2^. The threshold expression of *F*^2^ > σ(*F*^2^) is used only for calculating *R*-factors(gt) *etc*. and is not relevant to the choice of reflections for refinement. *R*-factors based on *F*^2^ are statistically about twice as large as those based on *F*, and *R*- factors based on ALL data will be even larger.

### Fractional atomic coordinates and isotropic or equivalent isotropic displacement parameters (Å^2^)

**Table:**

	*x*	*y*	*z*	*U*_iso_*/*U*_eq_	Occ. (<1)
C1	0.2795 (13)	0.643 (2)	0.395 (2)	0.051 (3)	0.545 (5)
C2	0.2648 (4)	0.8003 (10)	0.3360 (8)	0.063 (3)	0.545 (5)
H2	0.2407	0.8058	0.2482	0.076*	0.545 (5)
C3	0.2855 (4)	0.9506 (10)	0.4058 (8)	0.071 (3)	0.545 (5)
H3	0.2736	1.0559	0.3650	0.085*	0.545 (5)
C4	0.3239 (4)	0.9437 (10)	0.5365 (8)	0.074 (3)	0.545 (5)
H4	0.3377	1.0443	0.5832	0.089*	0.545 (5)
C5	0.3416 (4)	0.7863 (10)	0.5975 (8)	0.060 (2)	0.545 (5)
H5	0.3673	0.7816	0.6850	0.072*	0.545 (5)
C6	0.3209 (4)	0.6359 (10)	0.5277 (8)	0.053 (3)	0.545 (5)
C7	0.25776 (17)	0.4651 (5)	0.3147 (3)	0.0453 (18)	0.545 (5)
C8	0.18828 (17)	0.4355 (5)	0.2129 (3)	0.0410 (15)	0.545 (5)
C9	0.13446 (17)	0.5029 (5)	0.2303 (3)	0.0405 (13)	0.545 (5)
H9	0.1428	0.5752	0.2984	0.049*	0.545 (5)
C10	0.06822 (17)	0.4621 (5)	0.1459 (3)	0.0481 (16)	0.545 (5)
C11	0.05581 (17)	0.3540 (5)	0.0440 (3)	0.0542 (18)	0.545 (5)
C12	0.10963 (17)	0.2866 (5)	0.0266 (3)	0.0563 (17)	0.545 (5)
H12	0.1013	0.2143	−0.0415	0.068*	0.545 (5)
C13	0.17587 (17)	0.3274 (5)	0.1111 (3)	0.0523 (16)	0.545 (5)
H13	0.2119	0.2823	0.0994	0.063*	0.545 (5)
C14	0.0072 (3)	0.5223 (14)	0.1698 (8)	0.084 (3)	0.545 (5)
H14A	0.0233	0.5950	0.2432	0.126*	0.545 (5)
H14B	−0.0151	0.4244	0.1838	0.126*	0.545 (5)
H14C	−0.0243	0.5845	0.0973	0.126*	0.545 (5)
C15	−0.0137 (4)	0.3091 (12)	−0.0427 (7)	0.061 (2)	0.545 (5)
H15A	−0.0443	0.3659	−0.0170	0.091*	0.545 (5)
H15B	−0.0195	0.1875	−0.0411	0.091*	0.545 (5)
H15C	−0.0229	0.3441	−0.1271	0.091*	0.545 (5)
C16	0.3323 (3)	0.4755 (8)	0.5804 (6)	0.062 (3)	0.545 (5)
C17	0.4048 (3)	0.4417 (8)	0.6786 (6)	0.052 (2)	0.545 (5)
C18	0.4540 (3)	0.5214 (8)	0.6532 (6)	0.073 (3)	0.545 (5)
H18	0.4413	0.5945	0.5840	0.088*	0.545 (5)
C19	0.5223 (3)	0.4917 (8)	0.7311 (6)	0.082 (3)	0.545 (5)
H19	0.5552	0.5450	0.7141	0.098*	0.545 (5)
C20	0.5413 (3)	0.3824 (8)	0.8345 (6)	0.099 (4)	0.545 (5)
C21	0.4921 (3)	0.3028 (8)	0.8599 (6)	0.097 (4)	0.545 (5)
H21	0.5048	0.2297	0.9291	0.116*	0.545 (5)
C22	0.4238 (3)	0.3324 (8)	0.7820 (6)	0.088 (4)	0.545 (5)
H22	0.3909	0.2791	0.7990	0.105*	0.545 (5)
C23	0.6593 (4)	0.3804 (18)	0.9282 (15)	0.189 (7)	0.545 (5)
H23A	0.6566	0.3648	0.8450	0.284*	0.545 (5)
H23B	0.6976	0.3188	0.9890	0.284*	0.545 (5)
H23C	0.6643	0.4998	0.9492	0.284*	0.545 (5)
O1	0.3020 (6)	0.3679 (13)	0.3246 (14)	0.058 (3)	0.545 (5)
O2	0.2898 (11)	0.365 (3)	0.566 (2)	0.064 (4)	0.545 (5)
O3	0.6019 (4)	0.3206 (15)	0.9301 (9)	0.153 (4)	0.545 (5)
C1'	0.3153 (7)	0.6357 (14)	0.5030 (13)	0.054 (3)	0.455 (5)
C2'	0.3332 (8)	0.795 (2)	0.5636 (13)	0.073 (4)	0.455 (5)
H2'	0.3612	0.7994	0.6503	0.087*	0.455 (5)
C3'	0.3099 (7)	0.945 (2)	0.4963 (14)	0.087 (4)	0.455 (5)
H3'	0.3205	1.0514	0.5352	0.104*	0.455 (5)
C4'	0.2702 (8)	0.927 (2)	0.3688 (15)	0.082 (4)	0.455 (5)
H4'	0.2568	1.0251	0.3196	0.099*	0.455 (5)
C5'	0.2495 (8)	0.776 (2)	0.3109 (13)	0.073 (3)	0.455 (5)
H5'	0.2174	0.7731	0.2265	0.088*	0.455 (5)
C6'	0.2754 (15)	0.626 (2)	0.375 (2)	0.053 (3)	0.455 (5)
C7'	0.3402 (3)	0.4675 (9)	0.5975 (6)	0.047 (2)	0.455 (5)
C8'	0.4154 (3)	0.4348 (9)	0.6869 (6)	0.061 (3)	0.455 (5)
C9'	0.4712 (3)	0.4976 (9)	0.6739 (6)	0.0469 (19)	0.455 (5)
H9'	0.4649	0.5677	0.6056	0.056*	0.455 (5)
C10'	0.5363 (3)	0.4558 (9)	0.7629 (6)	0.0415 (18)	0.455 (5)
C11'	0.5457 (3)	0.3511 (9)	0.8650 (6)	0.0474 (19)	0.455 (5)
C12'	0.4900 (3)	0.2882 (9)	0.8780 (6)	0.073 (3)	0.455 (5)
H12'	0.4963	0.2182	0.9463	0.088*	0.455 (5)
C13'	0.4248 (3)	0.3301 (9)	0.7889 (6)	0.067 (3)	0.455 (5)
H13'	0.3875	0.2880	0.7976	0.080*	0.455 (5)
C14'	0.5888 (3)	0.5345 (13)	0.7310 (8)	0.064 (2)	0.455 (5)
H14D	0.6331	0.5034	0.7942	0.096*	0.455 (5)
H14E	0.5842	0.6567	0.7286	0.096*	0.455 (5)
H14F	0.5829	0.4939	0.6496	0.096*	0.455 (5)
C15'	0.6212 (5)	0.318 (2)	0.9610 (10)	0.075 (3)	0.455 (5)
H15D	0.6505	0.3750	0.9321	0.112*	0.455 (5)
H15E	0.6302	0.1976	0.9672	0.112*	0.455 (5)
H15F	0.6293	0.3620	1.0428	0.112*	0.455 (5)
C16'	0.2607 (3)	0.4816 (7)	0.3035 (5)	0.055 (2)	0.455 (5)
C17'	0.1879 (3)	0.4505 (7)	0.2433 (5)	0.061 (2)	0.455 (5)
C18'	0.1400 (3)	0.5283 (7)	0.2734 (5)	0.086 (3)	0.455 (5)
H18'	0.1539	0.5978	0.3447	0.103*	0.455 (5)
C19'	0.0713 (3)	0.5023 (7)	0.1969 (5)	0.088 (3)	0.455 (5)
H19'	0.0393	0.5544	0.2170	0.105*	0.455 (5)
C20'	0.0505 (3)	0.3985 (7)	0.0903 (5)	0.067 (2)	0.455 (5)
C21'	0.0984 (3)	0.3206 (7)	0.0602 (5)	0.072 (3)	0.455 (5)
H21'	0.0845	0.2512	−0.0111	0.086*	0.455 (5)
C22'	0.1671 (3)	0.3466 (7)	0.1367 (5)	0.070 (2)	0.455 (5)
H22'	0.1992	0.2946	0.1166	0.084*	0.455 (5)
C23'	−0.0429 (8)	0.293 (2)	−0.0741 (13)	0.147 (7)	0.455 (5)
H23D	−0.0473	0.3639	−0.1441	0.220*	0.455 (5)
H23E	−0.0861	0.2433	−0.0916	0.220*	0.455 (5)
H23F	−0.0106	0.2040	−0.0620	0.220*	0.455 (5)
O1'	0.2947 (14)	0.375 (4)	0.593 (3)	0.061 (4)	0.455 (5)
O2'	0.3057 (9)	0.378 (2)	0.322 (3)	0.097 (5)	0.455 (5)
O3'	−0.0204 (3)	0.3942 (8)	0.0374 (6)	0.0958 (19)	0.455 (5)

### Atomic displacement parameters (Å^2^)

**Table:**

	*U*^11^	*U*^22^	*U*^33^	*U*^12^	*U*^13^	*U*^23^
C1	0.049 (5)	0.035 (4)	0.066 (6)	0.004 (3)	0.023 (4)	−0.009 (3)
C2	0.060 (5)	0.038 (4)	0.079 (6)	−0.009 (4)	0.018 (4)	0.004 (4)
C3	0.071 (6)	0.026 (3)	0.096 (7)	−0.004 (4)	0.019 (5)	0.004 (5)
C4	0.072 (6)	0.035 (4)	0.089 (7)	−0.002 (4)	0.012 (5)	−0.006 (4)
C5	0.065 (5)	0.037 (4)	0.073 (5)	−0.006 (3)	0.027 (4)	−0.010 (4)
C6	0.059 (4)	0.048 (5)	0.056 (4)	−0.005 (3)	0.028 (4)	−0.002 (3)
C7	0.050 (4)	0.030 (3)	0.055 (4)	0.005 (3)	0.022 (3)	0.005 (3)
C8	0.048 (3)	0.037 (3)	0.040 (3)	0.010 (3)	0.021 (2)	0.002 (2)
C9	0.040 (3)	0.043 (3)	0.039 (3)	0.006 (2)	0.018 (2)	0.002 (2)
C10	0.063 (4)	0.041 (3)	0.042 (3)	0.002 (3)	0.025 (3)	−0.006 (3)
C11	0.074 (4)	0.040 (3)	0.053 (4)	0.010 (3)	0.032 (3)	−0.002 (3)
C12	0.077 (4)	0.053 (4)	0.056 (3)	−0.013 (3)	0.045 (3)	−0.018 (3)
C13	0.069 (4)	0.038 (3)	0.069 (4)	−0.004 (3)	0.047 (3)	−0.015 (3)
C14	0.068 (5)	0.081 (6)	0.092 (6)	0.007 (4)	0.026 (4)	−0.004 (5)
C15	0.076 (5)	0.054 (4)	0.055 (4)	−0.007 (4)	0.031 (4)	−0.013 (3)
C16	0.049 (4)	0.057 (5)	0.064 (4)	0.008 (4)	0.011 (3)	−0.003 (4)
C17	0.041 (3)	0.047 (4)	0.068 (5)	0.012 (3)	0.025 (3)	−0.009 (4)
C18	0.033 (3)	0.071 (5)	0.102 (5)	0.011 (3)	0.018 (3)	−0.021 (4)
C19	0.034 (3)	0.080 (6)	0.110 (6)	0.004 (4)	0.013 (4)	−0.017 (5)
C20	0.052 (4)	0.081 (6)	0.119 (6)	0.019 (4)	−0.003 (4)	−0.025 (5)
C21	0.088 (6)	0.069 (6)	0.096 (6)	0.032 (5)	0.006 (5)	0.013 (5)
C22	0.069 (6)	0.074 (6)	0.089 (6)	0.028 (5)	0.007 (5)	0.005 (6)
C23	0.067 (5)	0.186 (11)	0.219 (12)	0.027 (7)	−0.025 (7)	−0.041 (10)
O1	0.056 (5)	0.037 (3)	0.064 (5)	0.007 (3)	0.011 (4)	−0.009 (3)
O2	0.057 (5)	0.051 (5)	0.069 (10)	−0.009 (3)	0.014 (6)	0.002 (5)
O3	0.088 (5)	0.135 (6)	0.160 (7)	0.023 (5)	−0.014 (5)	−0.061 (5)
C1'	0.064 (5)	0.036 (5)	0.066 (5)	0.008 (4)	0.031 (4)	−0.009 (3)
C2'	0.073 (6)	0.063 (7)	0.061 (6)	−0.010 (5)	0.009 (5)	−0.005 (5)
C3'	0.079 (7)	0.050 (6)	0.085 (7)	−0.004 (5)	−0.006 (6)	−0.011 (5)
C4'	0.080 (7)	0.051 (6)	0.090 (7)	−0.005 (5)	0.015 (5)	0.002 (5)
C5'	0.068 (6)	0.054 (5)	0.070 (6)	−0.019 (4)	0.006 (4)	−0.004 (4)
C6'	0.041 (5)	0.046 (5)	0.068 (7)	0.002 (4)	0.021 (5)	−0.001 (4)
C7'	0.068 (5)	0.040 (5)	0.044 (4)	0.002 (4)	0.035 (3)	−0.005 (3)
C8'	0.057 (4)	0.057 (5)	0.065 (5)	0.004 (4)	0.025 (4)	−0.009 (5)
C9'	0.047 (4)	0.042 (4)	0.050 (4)	0.007 (3)	0.020 (3)	0.004 (3)
C10'	0.033 (3)	0.038 (4)	0.050 (4)	0.009 (3)	0.016 (3)	0.002 (3)
C11'	0.054 (4)	0.048 (4)	0.043 (4)	0.008 (3)	0.024 (3)	0.003 (3)
C12'	0.079 (6)	0.075 (6)	0.069 (5)	−0.005 (5)	0.037 (5)	0.010 (5)
C13'	0.061 (6)	0.081 (7)	0.068 (6)	−0.003 (5)	0.038 (5)	−0.002 (6)
C14'	0.050 (4)	0.066 (6)	0.088 (6)	−0.009 (4)	0.042 (4)	−0.002 (5)
C15'	0.061 (6)	0.100 (7)	0.049 (5)	−0.036 (5)	0.012 (4)	−0.001 (5)
C16'	0.041 (4)	0.055 (5)	0.072 (5)	0.006 (4)	0.029 (4)	0.018 (4)
C17'	0.061 (5)	0.063 (4)	0.052 (4)	−0.001 (4)	0.017 (3)	0.002 (3)
C18'	0.064 (5)	0.085 (6)	0.101 (6)	0.008 (4)	0.030 (4)	0.002 (5)
C19'	0.071 (5)	0.087 (6)	0.111 (6)	−0.002 (4)	0.047 (4)	0.015 (5)
C20'	0.047 (3)	0.061 (5)	0.085 (5)	−0.006 (4)	0.021 (4)	0.015 (4)
C21'	0.073 (5)	0.065 (5)	0.061 (4)	0.003 (4)	0.014 (4)	0.000 (4)
C22'	0.067 (4)	0.073 (5)	0.070 (4)	0.010 (4)	0.029 (4)	0.006 (4)
C23'	0.110 (11)	0.137 (11)	0.118 (10)	−0.048 (9)	−0.018 (7)	0.033 (8)
O1'	0.058 (5)	0.063 (6)	0.055 (8)	−0.023 (4)	0.017 (5)	−0.003 (5)
O2'	0.067 (7)	0.107 (9)	0.127 (10)	0.016 (7)	0.053 (7)	0.011 (8)
O3'	0.092 (4)	0.093 (4)	0.093 (4)	−0.014 (3)	0.032 (3)	−0.012 (3)

### Geometric parameters (Å, º)

**Table:**

C1—C2	1.38 (2)	C1'—C6'	1.37 (2)
C1—C6	1.42 (2)	C1'—C2'	1.401 (15)
C1—C7	1.628 (10)	C1'—C7'	1.651 (11)
C2—C3	1.3900	C2'—C3'	1.380 (16)
C2—H2	0.9300	C2'—H2'	0.9300
C3—C4	1.3900	C3'—C4'	1.369 (13)
C3—H3	0.9300	C3'—H3'	0.9300
C4—C5	1.3901	C4'—C5'	1.333 (16)
C4—H4	0.9300	C4'—H4'	0.9300
C5—C6	1.3900	C5'—C6'	1.38 (3)
C5—H5	0.9300	C5'—H5'	0.9300
C6—C16	1.372 (9)	C6'—C16'	1.355 (12)
C7—O1	1.198 (11)	C7'—O1'	1.212 (15)
C7—C8	1.4856	C7'—C8'	1.5405
C8—C9	1.3900	C8'—C9'	1.3899
C8—C13	1.3900	C8'—C13'	1.3901
C9—C10	1.3900	C9'—C10'	1.3901
C9—H9	0.9300	C9'—H9'	0.9300
C10—C11	1.3901	C10'—C11'	1.3900
C10—C14	1.561 (7)	C10'—C14'	1.495 (7)
C11—C12	1.3900	C11'—C12'	1.3901
C11—C15	1.457 (7)	C11'—C15'	1.563 (8)
C12—C13	1.3900	C12'—C13'	1.3899
C12—H12	0.9300	C12'—H12'	0.9300
C13—H13	0.9300	C13'—H13'	0.9300
C14—H14A	0.9600	C14'—H14D	0.9600
C14—H14B	0.9600	C14'—H14E	0.9600
C14—H14C	0.9600	C14'—H14F	0.9600
C15—H15A	0.9600	C15'—H15D	0.9600
C15—H15B	0.9600	C15'—H15E	0.9600
C15—H15C	0.9600	C15'—H15F	0.9600
C16—O2	1.231 (14)	C16'—O2'	1.220 (15)
C16—C17	1.5239	C16'—C17'	1.4562
C17—C18	1.3899	C17'—C18'	1.3899
C17—C22	1.3900	C17'—C22'	1.3901
C18—C19	1.3901	C18'—C19'	1.3900
C18—H18	0.9300	C18'—H18'	0.9300
C19—C20	1.3900	C19'—C20'	1.3900
C19—H19	0.9300	C19'—H19'	0.9300
C20—C21	1.3902	C20'—C21'	1.3901
C20—O3	1.396 (7)	C20'—O3'	1.402 (7)
C21—C22	1.3899	C21'—C22'	1.3900
C21—H21	0.9300	C21'—H21'	0.9300
C22—H22	0.9300	C22'—H22'	0.9300
C23—O3	1.355 (9)	C23'—O3'	1.418 (10)
C23—H23A	0.9600	C23'—H23D	0.9600
C23—H23B	0.9600	C23'—H23E	0.9600
C23—H23C	0.9600	C23'—H23F	0.9600
			
C2—C1—C6	118.8 (10)	C3'—C2'—C1'	120.9 (10)
C2—C1—C7	121.6 (13)	C3'—C2'—H2'	119.6
C6—C1—C7	119.3 (14)	C1'—C2'—H2'	119.6
C1—C2—C3	120.9 (6)	C4'—C3'—C2'	115.8 (14)
C1—C2—H2	119.5	C4'—C3'—H3'	122.1
C3—C2—H2	119.5	C2'—C3'—H3'	122.1
C2—C3—C4	120.0	C5'—C4'—C3'	124.0 (15)
C2—C3—H3	120.0	C5'—C4'—H4'	118.0
C4—C3—H3	120.0	C3'—C4'—H4'	118.0
C3—C4—C5	120.0	C4'—C5'—C6'	120.6 (12)
C3—C4—H4	120.0	C4'—C5'—H5'	119.7
C5—C4—H4	120.0	C6'—C5'—H5'	119.7
C6—C5—C4	120.0	C16'—C6'—C1'	126 (2)
C6—C5—H5	120.0	C16'—C6'—C5'	116.3 (17)
C4—C5—H5	120.0	C1'—C6'—C5'	117.4 (14)
C16—C6—C5	124.0 (5)	O1'—C7'—C8'	123.2 (16)
C16—C6—C1	115.5 (9)	O1'—C7'—C1'	114.9 (17)
C5—C6—C1	120.1 (7)	C8'—C7'—C1'	121.9 (5)
O1—C7—C8	118.8 (5)	C9'—C8'—C13'	120.0
O1—C7—C1	117.8 (11)	C9'—C8'—C7'	126.9
C8—C7—C1	122.4 (9)	C13'—C8'—C7'	113.1
C9—C8—C13	120.0	C8'—C9'—C10'	120.0
C9—C8—C7	117.5	C8'—C9'—H9'	120.0
C13—C8—C7	122.0	C10'—C9'—H9'	120.0
C8—C9—C10	120.0	C11'—C10'—C9'	120.0
C8—C9—H9	120.0	C11'—C10'—C14'	128.4 (4)
C10—C9—H9	120.0	C9'—C10'—C14'	111.6 (4)
C9—C10—C11	120.0	C10'—C11'—C12'	120.0
C9—C10—C14	121.1 (4)	C10'—C11'—C15'	115.2 (5)
C11—C10—C14	118.7 (4)	C12'—C11'—C15'	124.7 (5)
C12—C11—C10	120.0	C13'—C12'—C11'	120.0
C12—C11—C15	120.2 (3)	C13'—C12'—H12'	120.0
C10—C11—C15	119.7 (3)	C11'—C12'—H12'	120.0
C11—C12—C13	120.0	C12'—C13'—C8'	120.0
C11—C12—H12	120.0	C12'—C13'—H13'	120.0
C13—C12—H12	120.0	C8'—C13'—H13'	120.0
C12—C13—C8	120.0	C10'—C14'—H14D	109.5
C12—C13—H13	120.0	C10'—C14'—H14E	109.5
C8—C13—H13	120.0	H14D—C14'—H14E	109.5
C10—C14—H14A	109.5	C10'—C14'—H14F	109.5
C10—C14—H14B	109.5	H14D—C14'—H14F	109.5
H14A—C14—H14B	109.5	H14E—C14'—H14F	109.5
C10—C14—H14C	109.5	C11'—C15'—H15D	109.5
H14A—C14—H14C	109.5	C11'—C15'—H15E	109.5
H14B—C14—H14C	109.5	H15D—C15'—H15E	109.5
C11—C15—H15A	109.5	C11'—C15'—H15F	109.5
C11—C15—H15B	109.5	H15D—C15'—H15F	109.5
H15A—C15—H15B	109.5	H15E—C15'—H15F	109.5
C11—C15—H15C	109.5	O2'—C16'—C6'	119.0 (16)
H15A—C15—H15C	109.5	O2'—C16'—C17'	127.4 (10)
H15B—C15—H15C	109.5	C6'—C16'—C17'	110.5 (14)
O2—C16—C6	127.3 (14)	C18'—C17'—C22'	120.0
O2—C16—C17	117.3 (12)	C18'—C17'—C16'	126.6
C6—C16—C17	114.9 (4)	C22'—C17'—C16'	112.9
C18—C17—C22	120.0	C17'—C18'—C19'	120.0
C18—C17—C16	114.4	C17'—C18'—H18'	120.0
C22—C17—C16	125.4	C19'—C18'—H18'	120.0
C17—C18—C19	120.0	C20'—C19'—C18'	120.0
C17—C18—H18	120.0	C20'—C19'—H19'	120.0
C19—C18—H18	120.0	C18'—C19'—H19'	120.0
C20—C19—C18	120.0	C19'—C20'—C21'	120.0
C20—C19—H19	120.0	C19'—C20'—O3'	105.9 (3)
C18—C19—H19	120.0	C21'—C20'—O3'	134.1 (3)
C19—C20—C21	120.0	C22'—C21'—C20'	120.0
C19—C20—O3	136.8 (6)	C22'—C21'—H21'	120.0
C21—C20—O3	103.2 (6)	C20'—C21'—H21'	120.0
C22—C21—C20	120.0	C21'—C22'—C17'	120.0
C22—C21—H21	120.0	C21'—C22'—H22'	120.0
C20—C21—H21	120.0	C17'—C22'—H22'	120.0
C21—C22—C17	120.0	O3'—C23'—H23D	109.5
C21—C22—H22	120.0	O3'—C23'—H23E	109.5
C17—C22—H22	120.0	H23D—C23'—H23E	109.5
C23—O3—C20	115.6 (9)	O3'—C23'—H23F	109.5
C6'—C1'—C2'	120.6 (12)	H23D—C23'—H23F	109.5
C6'—C1'—C7'	123.7 (13)	H23E—C23'—H23F	109.5
C2'—C1'—C7'	115.5 (10)	C20'—O3'—C23'	106.9 (8)
			
C6—C1—C2—C3	−5 (3)	C6'—C1'—C2'—C3'	1 (2)
C7—C1—C2—C3	−178.1 (13)	C7'—C1'—C2'—C3'	−173.8 (12)
C1—C2—C3—C4	2.6 (14)	C1'—C2'—C3'—C4'	−1.4 (17)
C2—C3—C4—C5	0.0	C2'—C3'—C4'—C5'	5.3 (15)
C3—C4—C5—C6	0.0	C3'—C4'—C5'—C6'	−9 (3)
C4—C5—C6—C16	−175.7 (6)	C2'—C1'—C6'—C16'	172 (2)
C4—C5—C6—C1	−2.5 (14)	C7'—C1'—C6'—C16'	−13 (4)
C2—C1—C6—C16	178.8 (16)	C2'—C1'—C6'—C5'	−5 (3)
C7—C1—C6—C16	−8 (3)	C7'—C1'—C6'—C5'	169.8 (17)
C2—C1—C6—C5	5 (3)	C4'—C5'—C6'—C16'	−168.9 (19)
C7—C1—C6—C5	178.2 (12)	C4'—C5'—C6'—C1'	9 (4)
C2—C1—C7—O1	119 (2)	C6'—C1'—C7'—O1'	−55 (3)
C6—C1—C7—O1	−54 (3)	C2'—C1'—C7'—O1'	120.1 (19)
C2—C1—C7—C8	−49 (3)	C6'—C1'—C7'—C8'	126.6 (18)
C6—C1—C7—C8	138.5 (15)	C2'—C1'—C7'—C8'	−58.3 (9)
O1—C7—C8—C9	156.0 (9)	O1'—C7'—C8'—C9'	159.0 (19)
C1—C7—C8—C9	−36.2 (12)	C1'—C7'—C8'—C9'	−22.7 (7)
O1—C7—C8—C13	−16.1 (9)	O1'—C7'—C8'—C13'	−20.8 (19)
C1—C7—C8—C13	151.7 (12)	C1'—C7'—C8'—C13'	157.4 (7)
C13—C8—C9—C10	0.0	C13'—C8'—C9'—C10'	0.0
C7—C8—C9—C10	−172.3	C7'—C8'—C9'—C10'	−179.8
C8—C9—C10—C11	0.0	C8'—C9'—C10'—C11'	0.0
C8—C9—C10—C14	174.4 (5)	C8'—C9'—C10'—C14'	179.4 (5)
C9—C10—C11—C12	0.0	C9'—C10'—C11'—C12'	0.0
C14—C10—C11—C12	−174.5 (5)	C14'—C10'—C11'—C12'	−179.3 (6)
C9—C10—C11—C15	178.8 (5)	C9'—C10'—C11'—C15'	−178.0 (8)
C14—C10—C11—C15	4.3 (6)	C14'—C10'—C11'—C15'	2.7 (8)
C10—C11—C12—C13	0.0	C10'—C11'—C12'—C13'	0.0
C15—C11—C12—C13	−178.8 (5)	C15'—C11'—C12'—C13'	177.8 (8)
C11—C12—C13—C8	0.0	C11'—C12'—C13'—C8'	0.0
C9—C8—C13—C12	0.0	C9'—C8'—C13'—C12'	0.0
C7—C8—C13—C12	171.9	C7'—C8'—C13'—C12'	179.8
C5—C6—C16—O2	124.1 (16)	C1'—C6'—C16'—O2'	−42 (4)
C1—C6—C16—O2	−49 (2)	C5'—C6'—C16'—O2'	135 (2)
C5—C6—C16—C17	−48.6 (6)	C1'—C6'—C16'—C17'	119 (3)
C1—C6—C16—C17	138.0 (13)	C5'—C6'—C16'—C17'	−63 (3)
O2—C16—C17—C18	151.6 (14)	O2'—C16'—C17'—C18'	145.8 (17)
C6—C16—C17—C18	−35.0 (6)	C6'—C16'—C17'—C18'	−14.0 (14)
O2—C16—C17—C22	−23.9 (14)	O2'—C16'—C17'—C22'	−42.1 (17)
C6—C16—C17—C22	149.5 (6)	C6'—C16'—C17'—C22'	158.1 (14)
C22—C17—C18—C19	0.0	C22'—C17'—C18'—C19'	0.0
C16—C17—C18—C19	−175.8	C16'—C17'—C18'—C19'	171.6
C17—C18—C19—C20	0.0	C17'—C18'—C19'—C20'	0.0
C18—C19—C20—C21	0.0	C18'—C19'—C20'—C21'	0.0
C18—C19—C20—O3	179.0 (8)	C18'—C19'—C20'—O3'	178.0 (4)
C19—C20—C21—C22	0.0	C19'—C20'—C21'—C22'	0.0
O3—C20—C21—C22	−179.3 (6)	O3'—C20'—C21'—C22'	−177.4 (5)
C20—C21—C22—C17	0.0	C20'—C21'—C22'—C17'	0.0
C18—C17—C22—C21	0.0	C18'—C17'—C22'—C21'	0.0
C16—C17—C22—C21	175.3	C16'—C17'—C22'—C21'	−172.7
C19—C20—O3—C23	1.4 (15)	C19'—C20'—O3'—C23'	178.1 (8)
C21—C20—O3—C23	−179.5 (10)	C21'—C20'—O3'—C23'	−4.2 (11)

### Hydrogen-bond geometry (Å, º)

**Table:**

*D*—H···*A*	*D*—H	H···*A*	*D*···*A*	*D*—H···*A*
C23—H23*B*···O2^i^	0.96	2.32	3.23 (3)	159

Symmetry code: (i) *x*+1/2, −*y*+1/2, *z*+1/2.
